# Long-Term Impact of Interprofessional Medical Mission Service Trips in Sierra Leone

**DOI:** 10.3389/fmed.2021.742406

**Published:** 2021-09-27

**Authors:** Yen Tran, Jennie Jarrett, Scott Gardner, James Fernando, Mark Milliron, Lisa Hong

**Affiliations:** ^1^Department of Pharmacy Practice, Loma Linda University School of Pharmacy, Loma Linda, CA, United States; ^2^College of Pharmacy, University of Illinois at Chicago, Chicago, IL, United States; ^3^Department of Physician Assistants, Kettering College, Kettering, OH, United States; ^4^Adventist Health System Waterloo Hospital, Waterloo, Sierra Leone; ^5^Department of Physician Assistant Sciences, Loma Linda University, Loma Linda, CA, United States

**Keywords:** global health, health education, medical missions, interprofessional, Africa

## Abstract

**Objective:** The purpose of this study was to evaluate the impact of capacity-building short-term mission service trips to Sierra Leone on local health education and perspectives.

**Methods:** This was a prospective, mixed-methods study. During three mission trips between June 2017 and December 2019, health professional students taught multiple locally selected patient care-related topics. Local staff completed knowledge questionnaires and were surveyed or interviewed on mission service impact along with the cultural competence of missionaries. Mission team members completed the Intercultural Effectiveness Scale (IES) and surveys to determine their cultural competence.

**Results:** After initial education, 90% passed the knowledge questionnaire with at least a 50% and the correct response rate was 57.9 vs. 66.7% after 6 months and 2.5 years, respectively (*p* = 0.40). Local staff ranked education/training as most valuable (84%) and highly desired (53%). Mean IES score and survey responses of both missionaries and local staff rated mission team cultural competence as average.

**Conclusions:** Education-focused mission trips in Sierra Leone seem to have long-lasting benefits and a positive impact on local staff, though improved intercultural competence is needed.

## Introduction

International medical mission service trips can be valuable and transformational for health profession students. While the focus is often on personal and professional development of student missionaries, it is critical to make local needs a priority, establish sustainable partnerships, and balance student learning objectives with the needs of the communities they serve.

Short-term mission trips have long been criticized for their short-term impact and unintended negative consequences, including limited sustainability of services provided beyond the duration of the trip (1–5). Temporary free services by mission teams including those outside of their scope of practice with limited local-missionary collaboration may disrupt normal workflow, burden local staff, and create challenges for continuity and patient follow-up with health professionals (2, 6–10). Furthermore, lack of cultural competence and adequate understanding of local health concerns or health systems may result in miscommunication and inappropriate preventive care (2, 10).

An approach for medical missions that focuses on sharing skills and knowledge with local staff, rather than providing one-time medical services, is described to support local growth without hindering their communities (11–13). Published guidelines regarding the ethics involved in short-term global health experiences emphasize the obligation of providing long-term benefits including education of local healthcare professionals (14, 15).

Short-term mission trips to Sierra Leone organized by Loma Linda University (LLU) focus on the provision of training and education of local staff as it pertains to local health needs. Benefits of international service opportunities for missionary teams include strengthening clinical skills, improving critical reasoning in environments with limited resources, as well as improving cultural competence and inter professional communication (16–20). However, the impact of education provided by mission teams to local healthcare providers has not been as widely evaluated in the literature. The objective of this work is to evaluate the impact of short-term mission trips to Sierra Leone on local health knowledge, gain understanding of perspectives of local practitioners, compare local and missionary perspectives, and describe missionary cultural awareness.

## Methods

### Hospital, Team, and Activities

Waterloo Adventist Health System (AHS) in Waterloo, Sierra Leone is located between suburban outskirts of the capital and rural communities. AHS is a small, 40-bed general hospital with 4 clinical units (one outpatient clinic and three inpatient wards: adult men, adult women, and pediatric patients), an operating room, pharmacy, laboratory, and physiotherapy room. The hospital serves both urban and rural communities, seeing around 3,000 to 5,000 outpatients annually with an average daily inpatient census of 20. Collaboration between LLU and AHS originated from mission work of an LLU pharmacy student and his continued relationship with long-term missionaries serving in Sierra Leone.

Three teams from LLU, including up to four health professions, served this community in June 2017, December 2017, and December 2019 for 10 to 17 days (Table 1). The focus of each trip was education for local staff with the content comprising of disease management and various clinical skills. Educational content was determined in collaboration with local staff through preparatory meetings before each trip, based on their needs and interests. From these meetings, teaching plans were determined to provide local staff with knowledge and skills in the areas of hypertension (HTN), diabetes mellitus (DM), and antibiotic therapy, to support their clinical practice even in the absence of mission teams. A list of locally available medications was obtained and student missionaries used this list to prepare slides, which they practiced presenting and revised based on feedback given before the trip. Local administrators scheduled and announced lecture times to their staff. During the trips, in addition to delivering lectures to local staff, missionaries participated in daily inpatient rounds as well as outpatient and mobile clinics, all of which occurred regularly irrespective of their presence. New processes were introduced to improve hospital workflow and enhance patient safety, such as the numbering of patient beds and paper charts for more accurate patient identification and organized record keeping. Mission teams also created treatment protocols for local hospital staff, which included management of HTN, DM, and infectious diseases. Furthermore, the hospital had plans to build a new pharmacy and the first mission team helped design the building, layout, and workflow prior to construction.

**Table 1:** Mission teams.

	**Team 1**	**Team 2**	**Team 3**
	**(*n* = 5)**	**(*n* = 8)**	**(*n* = 12)**
**Travel Dates**	June 2017	December 2017	December 2019
**Female Gender-** ***n*** **(%)**	2 (40.0)	2 (25.0)	4 (33.3)
**Age-*****n*** **(%)**			
20–29	5 (100.0)	5 (62.5)	9 (75.0)
30–39	-	1 (12.5)	2 (16.7)
40–49	-	-	-
50–59	-	2 (25.0)	1 (8.3)
**Profession of Missionaries-*****n*** **(%)**			
Pharmacy Student	4 (80.0)	4 (50.0)	2 (16.7)
Professional Year 3	-	4 (50.0)	1 (8.3)
Professional Year 4	4 (80.0)	-	1 (8.3)
Physician Assistant Student	-	-	8 (66.7)
Professional Year 1	-	-	1 (8.3)
Professional Year 2	-	-	7 (58.3)
Pharmacist	1 (20.0)	1 (12.5)	1 (8.3)
Physician Assistant	-	1 (12.5)	1 (8.3)
Speech Pathologist	-	1 (12.5)	-
Chaplain	-	1 (12.5)	-

### Assessments and Surveys

This was an IRB-approved, prospective, mixed-methods study of local staff at AHS and health profession students from LLU who participated in medical missions from 2017 to 2019. Local staff performance on knowledge questionnaires was collected, missionaries completed a Qualtrics survey regarding their service along with a scale to measure intercultural readiness, and local clinicians were asked to complete an open-ended survey on paper while administrators were interviewed to obtain qualitative data.

### Knowledge Questionnaires

**Figure 1:**
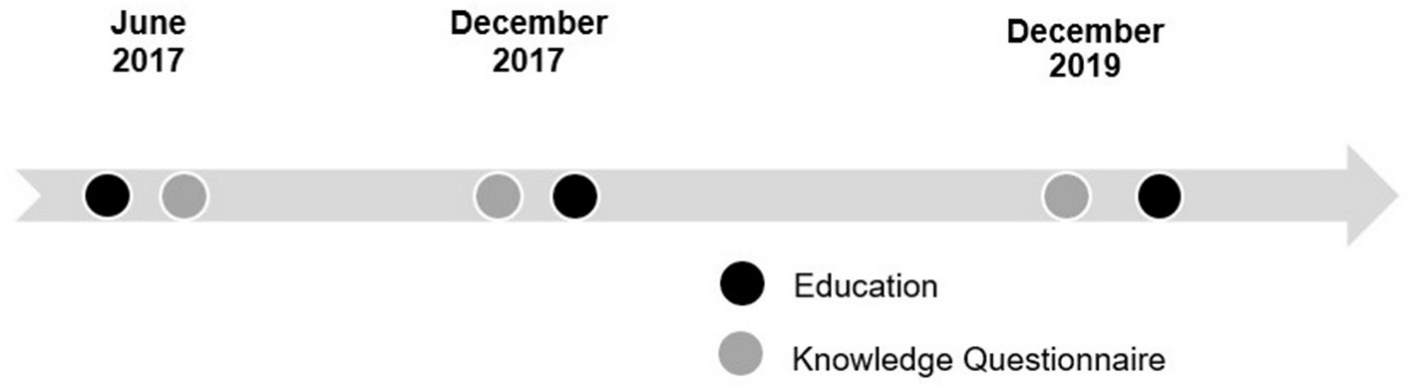
Timeline of education and local staff knowledge assessment.

Observation of local staff by mission teams during daily rounds or in clinic allowed them to learn and assess workflow and prescribing practices so that education could be appropriately adjusted to address local needs. In addition to HTN, DM, and antibiotic stewardship, lectures on medication safety were delivered to address system-level concerns observed. Missionaries utilized experiences from previous trips to modify teaching content for subsequent trips. Knowledge questionnaires were developed to assess learning objectives of each lecture and were given to local staff after the lectures in June 2017 (this served as the new educational baseline). The knowledge questionnaires were repeated at the beginning of the December 2017 and December 2019 trips, before any education was provided, to evaluate retention of information from previous trips (Figure 1). The 2017 knowledge questionnaires included 18 questions: 5 on HTN, 5 on DM, 3 on ABX, and 5 on medication safety. A total of 15 questions were on the December 2019 questionnaire (one less question in each of the latter 3 categories).

### Local Surveys

At the end of the second and third mission trips, local hospital administrators were interviewed, and local staff completed a 19-question survey, including open-ended questions about their perspectives on the value and impact of mission services. Questions in these interviews and surveys were reviewed and revised by a local administrator to ensure clarity and minimize misinterpretation. The questions aimed to evaluate whether education and services provided by mission teams met local expectations, were deemed valuable, or could be continued by local staff. Additionally, the survey aimed to assess whether mission teams were professionally and culturally appropriate.

### Missionary Surveys

An Intercultural Effectiveness Scale (IES) Survey (21) developed by Kozai Group was purchased from Aperian Global and administered to the December 2017 team to assess the level of cultural preparedness of participants before the trip. The survey evaluates three dimensions of intercultural effectiveness: continuous learning, interprofessional engagement, and hardiness of missionaries, which are detailed in Supplementary Table 1 (see Supplemental File).

The impact of mission service from the perspective of missionaries was also examined through pre-and post-trip surveys. These surveys also intended to provide a self-assessment of cultural competency and compare perceptions regarding interprofessional working relationships before and after the trip. Only cultural appropriateness and key take-away messages from the experience were analyzed for this paper.

### Statistical Analysis

Descriptive statistics with numbers and percentages for categorical data and means and standard deviations for continuous data were used to display the results. Chi-square and independent samples *t*-tests were used for the analysis of categorical and continuous data, respectively. A p-value of <0.05 was considered statistically significant. A schematic analysis was used to distill themes from open-ended questions and interview transcripts by two investigators individually and determinations were made by consensus.

## Results

The general demographics of local hospital staff are depicted in Table 2. During the first trip in June 2017, 20 local staff (57.1%) completed knowledge questionnaires immediately following lectures with pass rates (at least 50%) for each topic of 94, 95, 79, and 94% for HTN, DM, antibiotic therapy and medication safety, respectively, and 90% overall. There were 39 (88.6%) completed knowledge questionnaires in December 2017 and 36 (59.0%) in December 2019. A numerically higher rate of correct responses was seen in 2019 compared with the 2017 knowledge questionnaire (66.7 vs. 57.9%), but this did not reach statistical significance (*p* = 0.40). A similar trend was observed for each topic (Table 3). In December 2019, the average score was 71.1 with 78.8% of participants scoring higher than a passing score of 50%.

**Table 2:** Local staff demographics.

**Characteristic**	**June 2017**	**December 2017**	**December 2019**
	**(*n* = 35)**	**(*n* = 44)**	**(*n* = 61)**
**Female gender, n (%)**			
**Age**, ***n*** **(%)**	15 (42.9)	21 (47.7)	27 (44.1)
20–29	8 (22.9)	16 (36.4)	8 (13.1)
30–39	15 (42.9)	16 (35.4)	36 (59.0)
40–49	3 (8.6)	3 (6.8)	11 (18.0)
50–59	8 (22.9)	8 (18.2)	3 (4.9)
60–69	1 (2.9)	1 (2.3)	3 (4.9)
**Profession**, ***n*** **(%)**			
Nursing staff	19 (54.3)	25 (65.8)	21 (35.6)
Laboratory personnel	3 (8.6)	6 (15.8)	4 (6.8)
Providers	2 (5.7)	2 (5.3)	6 (10.2)
Physical therapy staff	2 (5.7)	2 (5.3)	2 (3.3)
Pharmacy personnel	1 (2.9)	1 (2.6)	2 (3.4)
Administration	5 (14.3)	5 (11.3)	7 (11.9)
Other^a^	3 (8.6)	3 (5.3)	15 (24.6)

a *Other professions include community resilience models, chaplains, maintenance, and security*.

**Table 3:** Results of knowledge questionnaires.

	**June 2017**	**December 2017**	**December 2019**	***p*-value**
	***(n* = 20)**	**(*n* = 39)**	**(*n* = 36)**	
**Overall pass rates, n (%)**	18 (90%)	–	28 (79%)	0.32
**% correct, mean ± SD**				
Overall	–	57.9 ± 29.3	66.7 ± 26.0	0.40
Hypertension questions	–	39.0 ± 28.4	52.2 ± 26.5	0.47
Diabetes questions	–	50.6 ± 29.1	54.2 ± 25.3	0.86
Antibiotic questions	–	73.1 ± 34.4	76.4 ± 9.8	0.91
Medication safety questions	–	81.4 ± 11.5	92.4 ± 6.2	0.15

Nineteen responses from the hospital staff survey on their perceptions of the missionaries and their value were recorded (Table 4). From open-ended questions that could include more than one answer, education and training were reported as most valuable (*n* = 16, 84%), highly desired (*n* = 10, 53%), and easy to continue (*n* = 15, 79%). Fifteen responses (79%) stated that services offered by missionaries met the local needs and were conducted in a similar manner locally. Sixteen responses (84%) expressed feeling appreciative of mission teams making changes to their practices and introducing new protocols. When addressing the cultural competency of missionaries, most responses (*n* = 10, 53%) indicated that locals were comfortable with missionary knowledge, respect for, and integration of cultural beliefs, and 14 respondents (74%) stated that the level of cultural awareness of the missionaries did not affect education and services provided.

**Table 4:** Local hospital survey and interview response summary.

**Survey Item**	***n* (%)**
**Professions**	
Nursing staff	16 (84)
Laboratory personnel	2 (11)
Pharmacy personnel	1 (5)
**Most valuable for the Adventist Health System** ^b^	
Education/teaching/training	16 (84)
Providing medical equipment/materials	3 (16)
Awarding certificates	3 (16)
**Services wanted** ^b^	
Education/teaching/training	10 (53)
Providing medical equipment/materials	3 (16)
Continuing what mission teams started	2 (11)
**Mission teams provided what was needed/wanted** ^b^	
Yes	15 (79)
Effort appreciated	3 (16)
**% of mission teams deemed helpful**	
25–50	3 (16)
50–75	9 (47)
75–100	7 (37)
**Services provided by mission teams compared to those same services provided by AHS** ^b^	
Same/almost same	15 (79)
Different	3 (16)
**How local staff felt about mission teams making changes to their practices** ^b^	
Good/Appreciative	16 (84)
Cannot compare	2 (11)
**How local staff felt about protocols mission teams introduced** ^b^	
Good/Appreciative	18 (95)
**Things mission teams did that was offensive** ^b^	
Nothing	13 (68)
Racism	2 (11)
Leaving	1 (5)
**Host and locals work well with mission teams^a^**	4.8 **±** 0.75
**Value of education vs. service from missionary teams**	
Equally valuable	15 (79)
Education more valuable	1 (5)
Service more valuable	0 (0)
**Skills and knowledge provided are easy to continue** ^b^	
Yes	15 (79)
Some	1 (5)
**Negative outcomes due to education or services provided** ^b^	
None	16 (84)
**Rating of missionary knowledge, respect for, and integration of local cultural beliefs**	
Did not demonstrate	0 (0)
Demonstrated some	1 (5)
Average	3 (16)
Felt comfortable with	10 (53)
Knew as if were their own	2 (11)
**How level of cultural awareness affected the education/healthcare services provided** ^b^	
No effect	14 (74)
Language barrier	1 (5)
**Education positively impacted level of care** ^a^	4.9 **±** 0.25
**Useful skills taught**	
CPR	8 (42)
Choking relief	5 (26)
Measurement of vitals	2 (11)
Waste management	4 (21)
**Suggestions for improvement of short-term mission trips** ^b^	
Extend stay	9 (47)
Visit again	10 (53)
Avoid racism	2 (11)
**Complaints about mission teams' services from local staff** ^b^	
No complaints	16 (84)
**Interview Quotes**	
“Successful [mission] teams use time to teach. The success [of a mission] is determined by how well the staff respond to the teaching.”	
“Hosting [a mission team] is not a lot of extra work. It is more than worth it and we are willing to provide what it takes.”	
“There is a commitment among staff to be committed to the job. They admire the [mission] team for willing to provide free services and that motivates them to work harder. They think they should do more so they have higher expectations.”	
“Some [staff] can perform what they have been taught and execute those skills.”	
“In discussions with human resources, every day, they want to see improved skills. Sometimes, [hospital staff] continue to teach what mission teams taught after morning worship.”	

*All values reported as n (%), unless otherwise specified*.

a *Data reported as mean ± standard deviation; Responses were based on 5-point Likert Scale with 1 = Strongly disagree and 5 = Strongly agree*.

b *Open-ended question themes were categorized through schematic analysis with some responses fitting into multiple categories*.

Administrators interviewed (*n* = 3) echoed the survey responses by their staff regarding mission service impact and sustainability (quotes in Table 4). In addition, they commented that development of relationships was valuable and mission trip success may be determined by the response of staff to the teaching. They also shared that local time and resources are limiting factors to achievement of expectations by mission teams. Regarding finances, time, and resources, it was mentioned that hosting mission teams does not add a lot of extra work and is more than worth the cost. Local administrators felt there had been good host-missionary communication and one administrator expressed that mission team presence raised expectations among their staff. When asked if there was a system in place to continue the implementation of education/services provided, it was discovered that even after missionaries have left, some of what was taught continues to be discussed after morning worship.

The IES survey was given to missionaries before the December 2017 mission trip with 7 responses recorded (Table 5). On a scale of 1 to 7, with 7 being the highest/most culturally competent, the team scored an average of 4.28 ± 1.98. The highest mean score of 4.71 ± 1.98 was recorded for self-awareness. The missionary team scored lowest on exploration (3.86 ± 1.57) and relationship development (3.86 ± 1.35).

**Table 5:** Summary of Intercultural Effectiveness Scale survey responses.

**Category**	**Score**
Continuous learning	4.28 ± 1.98
Self-awareness	4.71 ± 1.98
Exploration	3.86 ±1.57
Interprofessional engagement	4.14 ± 1.46
World orientation	4.43 ± 1.51
Relationship development	3.86 ± 1.35
Hardiness	4.14 ± 2.27
Positive regard	4.43 ± 1.72
Emotional resilience	4.43 ± 2.23
Overall	4.29 ± 1.98

*All data reported as mean ± standard deviation*.

Six missionary responses were recorded for the post-trip survey and for cultural competency, 33.3% were comfortable with their level of knowledge, respect for, and integration of local culture, 50% of the team rated themselves as average, and 16.7% felt they only demonstrated this to some degree. In a free-response question, the importance of communication was highlighted as a take-away from this interprofessional mission experience. One mission team member stated: “Communication is key. Learning the roles of other professions on your team is critical to success of the team. The knowledge base of the same professionals is different in Sierra Leone than in the United States and understanding the level of training at baseline and the level of training needed is important for successful education.” Another key takeaway written by one of the missionaries was: “the importance of being flexible–being able to work with limited resources and to deal with conflicting opinions.”

## Discussion

The mission service trips to Sierra Leone were conducted with emphasis on promoting the transfer of knowledge and clinical skills, determined through observation and collaboration with local practitioners in their daily responsibilities. This process allowed mission teams to identify areas of growth potential and improve current local practice in line with capacity-building best practice recommendations (22). Maintaining relationships with local staff, investing time in necessary preparatory work, as well as returning to the same site granted opportunities to modify educational content and accommodate local needs, ensuring both efficacy and continuity.

In evaluating performance of local staff on knowledge questionnaires, higher scores were seen in 2019 than in 2017; however, the increase was not statistically significant. After 2.5 years, approximately 80% of local staff passed the knowledge questionnaire (down only about 10% from June 2017), demonstrating considerable knowledge retention overall. Noticeably, the performance on medication safety questions remained highest among all topic areas assessed, supporting the sustainability of the education on this topic; one that is routinely applied to practice for most staff. To enhance continuity and incorporation into patient care, some knowledge shared with the staff, such as how to categorize hypertension, was printed on big posters around the hospital. However, hypertension questions had the lowest rate of correct responses compared with other topics. While there were fewer incorrect responses by 2019, still, <50% of local staff recognized that beta blockers are not the preferred antihypertensive medication. Beta blockers were previously used as the primary agents for hypertension management at the hospital. Despite local formulary review and multiple preparatory meetings before the trip, this usage of beta blockers was not discovered until the first mission team arrived in June 2017. Recognizing the teaching opportunity, the team modified the HTN lecture with additional slides to emphasize avoidance of beta blockers as first-line agents. Returning to the same hospital for follow-up may have contributed to improved local patient care, as evidenced by less utilization of beta-blockers in December 2017 and elimination of their use for hypertension management by December 2019. Termination of this practice may explain the low scores on questions regarding these medications and supports the effectiveness of education to optimize appropriate medication use and create long-term impact.

Findings from the knowledge questionnaires provided more detailed guidance for future education to meet the needs of local staff and bridge gaps in clinical knowledge. A confounder for knowledge of local staff was any unaccounted education from sources other than mission teams, such as their local health agencies. Another confounding factor that could not be controlled for was the large number of new staff (56%) who had not attended lectures from previous trips. The high overall pass rate, even with many new staff, underscores the degree of sustainability of our education potentially through current staff passing on knowledge gained to their new team members or use of educational posters. Though, this also calls attention to an area for growth in the realm of global health education as the ultimate goal is not just for knowledge to be sustained, but continuously improving.

The local interviews and surveys provide some qualitative data suggesting that services and education provided by missionaries were equally valued, met the needs of hospital staff, and paralleled what they desired. Mission teams worked closely with hospital administrators to prepare training materials and develop new protocols based on local needs, which likely contributed to the receptiveness and positive attitudes of local staff. When our teams first arrived, the hospital did not have a system in place to identify patients, which increased risk for safety errors. Implementation of bed numbering and paper charts may reduce medication errors and the continued use of this process over 2.5 years later illustrates the longevity of mission team efforts. To retain the benefits of hands-on training, mission teams continue to work with hospital administrators and providers longitudinally to establish re-certification processes where practical skills can be checked periodically in the absence of mission teams. The local survey responses highlight the success in providing clinical knowledge and useful skills that may be easily continued even after missionaries leave. The positive impact of mission teams is also supported by local wishes for mission teams to return.

Regarding cultural competency, most local staff felt comfortable with the cultural awareness of missionaries, which was comparable to missionary self-evaluations. However, the noting of racism by local staff reveals an important area for improvement as missionary teams must work to eliminate any sense of racism during their trips. A position paper by the American College of Physicians emphasizes sensitivity and respect for cultural differences as essential components of short-term global medical experiences (14). Mission teams that learn the local language, values, and beliefs will likely be well-received by the local community and be able to enhance the effectiveness of their efforts. Our findings underscore the necessity of including intercultural competence within the Accreditation Council for Pharmacy Education (ACPE) accreditation standards for schools of pharmacy that provide global health experiences. Intercultural and diversity effectiveness is crucial for having a positive impact, fostering positively strong relationships, and making sure that mission teams continue to be welcomed.

Some programs have utilized the IES as a tool to evaluate student intercultural competence and develop individualized plans for improvement, while others have incorporated this tool into their curriculum for cultural competency (23, 24). As a result, students from these programs expressed personal growth and indicated that these strategies helped them maximize their experiences. Utilization of the IES in our missionary cohort helped determine focus areas of cultural preparation prior to the mission. Whether this tool or another strategy is utilized, intercultural training should be incorporated into all curricula so that health profession graduates can effectively serve and interact with multicultural individuals both in the United States and abroad. Our institution has developed online modules to be completed prior to serving on medical mission trips to enhance culture awareness and cultural competence.

From the pre-post survey responses, we found that, qualitatively, mission service trips also provided learning opportunities for missionaries to think critically and work flexibly in situations of limited resources. When daily laboratory values and imaging were not readily available or extremely costly, missionaries had to rely on other aspects of care to guide their clinical decisions, such as conversing with patients and collaborating with other members of the healthcare team to conduct a review of systems and physical assessment for each patient. Effective communication is essential when working with health professionals of different disciplines and educational backgrounds. This experience highlights an opportunity to emphasize interprofessional education within the curriculum of every health profession program to optimize patient care and promote global health.

This study has several limitations. Given the inability to accommodate large teams, missionary sample size was limited. Furthermore, very few missionary team members completed the surveys. A large portion of local survey responses and results of knowledge questionnaires are representative of nursing staff, which is consistent with local staff demographics. Additionally, while pass rates and percent correct were recorded from the Dec 2019 knowledge questionnaires, only the former was available from June 2017 and only the latter was available from Dec 2017. This inconsistency in available measures precluded statistical comparison across all cohorts. However, we are confident that the initial educational programming in June 2017 was effective as the content in the knowledge questionnaires was selected in collaboration with local administrators based on areas deemed to be gaps in local staff knowledge and while answers were not collected, similar pre-lecture questions asked to engage the attendees could not be answered. Another limitation was the inability to administer the IES in a pre-post fashion and with each cohort due to the cost per survey.

## Conclusion

Short-term mission service trips to Sierra Leone potentially provided a positive long-term impact on local healthcare providers as evidenced by the retention and transfer of knowledge to new staff, improvement in hospital practice, and the desire of local staff for mission teams to return. In addition, these trips provide opportunities for students of various health professions to improve patient care through targeted education while learning to work with limited resources and exercising intercultural communication skills. This approach is mutually beneficial to mission teams and the communities they serve as knowledge and practical skills can continue to be utilized locally. More robust research is warranted to elucidate the long-lasting effects of short-term mission service on local healthcare providers, patients, and communities as well as the impact of these experiences on students when they become practitioners. This study adds value to a gap in global health experiences–making the most of the experience for both the local hosts and the visiting guests, which is helpful for missionaries to understand when planning medical mission trips.

## Data Availability Statement

The raw data supporting the conclusions of this article will be made available by the authors, without undue reservation.

## Ethics Statement

The studies involving human participants were reviewed and approved by Loma Linda University Investigational Review Board. Written informed consent for participation was not required for this study in accordance with the national legislation and the institutional requirements.

## Author Contributions

LH and JJ designed study surveys. Lecture content was created by YT, LH, and MM. Content of knowledge questionnaires was created by YT, LH, and other student missionaries. Data was collected and analyzed by YT and LH. The first draft of the manuscript was written by YT. SG, JF, and MM assisted with the logistical execution of the study (survey distribution, coordination of time, and distribution of knowledge questionnaires). SG and JF provided administrative support for the attendance of education. All authors revised manuscript.

## Conflict of Interest

The authors declare that the research was conducted in the absence of any commercial or financial relationships that could be construed as a potential conflict of interest.

## Publisher's Note

All claims expressed in this article are solely those of the authors and do not necessarily represent those of their affiliated organizations, or those of the publisher, the editors and the reviewers. Any product that may be evaluated in this article, or claim that may be made by its manufacturer, is not guaranteed or endorsed by the publisher.
