# Supplementary material for: Long-Term Impact of Interprofessional Medical Mission Service Trips in Sierra Leone
Source: Front Med (Lausanne). 2021 Sep 27;8:742406. doi: 10.3389/fmed.2021.742406 (PMC8502852; doi:10.3389/fmed.2021.742406)
Supplement: Supplementary file 1 [file Table_1.DOCX]

**Table S1. Descriptions of the IES elements**

| Dimension | Definition |
| --- | --- |
| Continuous Learning | |
| Self-Awareness | Measurement of self-awareness regarding personal strengths, weaknesses, behavioral tendencies and interprofessional style |
| Exploration | Assessment of one’s desire to learn new things and openness to new opportunities for change in behavior and perspective |
| Interpersonal Engagement | |
| World Orientation | Measurement of personal interest in other cultures and willingness to seek interactions with people who do not share the same values, beliefs or traditions |
| Relationship Development | Examination of one’s willingness to create and maintain relationships with people from other cultures |
| Hardiness | |
| Positive Regard | Self-assessment regarding degree of assuming the genuine good in people |
| Emotional Resilience | Measurement of one’s emotional strength and ability to recover from emotional and psychological stress |
